# (3-Benzoyl­phen­yl)(phen­yl)methanone

**DOI:** 10.1107/S1600536811033344

**Published:** 2011-08-27

**Authors:** Ahmed Raza Ahsraf, Zareen Akhter, Michael Bolte

**Affiliations:** a Department of Chemistry, Quaid-I-Azam University, Islamabad 45320, Pakistan; b Institut für Anorganische Chemie, J. W. Goethe-Universität Frankfurt, Max-von-Laue-Strasse 7, 60438 Frankfurt/Main, Germany

## Abstract

Mol­ecules of the title compound, C_20_H_14_O_2_, show approximate *C*_s_ symmetry with the approximate mirror plane perpendicular to the central ring. The torsion angles about the acyclic bonds are 30.05 (15) and 30.77 (15)° in one half compared to −36.62 (14) and −18.60 (15)° in the other half of the mol­ecule. The central aromatic ring makes dihedral angles of 47.78 (4) and 51.68 (3)° with the two terminal rings.

## Related literature

For background to diaryl­ketones, see: Olah (1964 ▶); Szmant (1989 ▶); March (1992 ▶). For the synthesis of benzoyl­benzene and its derivatives, see: Karrer *et al.* (2000 ▶); Kowalski *et al.* (2005 ▶). For its natural occurrence, see: Baggett *et al.* (2005 ▶); Chiang *et al.* (2003 ▶); Bernardi, *et al.* (2005 ▶); Kulanthaivel *et al.* (1993 ▶); Iijima *et al.* (2004 ▶). For applications of these compounds, see: Bohm *et al.* (2001 ▶); Chan *et al.* (2004 ▶); Bagheri *et al.* (2000 ▶); Husain *et al.* (2006 ▶).
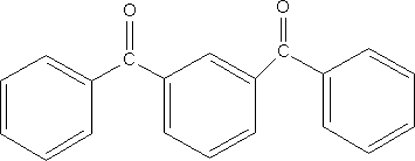


## Experimental

### 

#### Crystal data

C_20_H_14_O_2_
*M* *_r_* = 286.31
Orthorhombic, 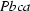

*a* = 16.2029 (5) Å
*b* = 7.8648 (4) Å
*c* = 22.8422 (8) Å
*V* = 2910.8 (2) Å^3^
*Z* = 8
Mo *K*α radiation
μ = 0.08 mm^−1^
*T* = 173 K
0.45 × 0.45 × 0.43 mm

#### Data collection

Stoe IPDS II two-circle diffractometer
40513 measured reflections
3653 independent reflections
3095 reflections with *I* > 2σ(*I*)
*R*_int_ = 0.050

#### Refinement

*R*[*F* ^2^ > 2σ(*F* ^2^)] = 0.037
*wR*(*F* ^2^) = 0.101
*S* = 1.05
3653 reflections
200 parameters
H-atom parameters constrained
Δρ_max_ = 0.28 e Å^−3^
Δρ_min_ = −0.15 e Å^−3^

### 

Data collection: *X-AREA* (Stoe & Cie, 2001 ▶); cell refinement: *X-AREA*; data reduction: *X-AREA*; program(s) used to solve structure: *SHELXS97* (Sheldrick, 2008 ▶); program(s) used to refine structure: *SHELXL97* (Sheldrick, 2008 ▶); molecular graphics: *XP* in *SHELXTL* (Sheldrick, 2008 ▶); software used to prepare material for publication: *SHELXL97*.

## Supplementary Material

Crystal structure: contains datablock(s) I, global. DOI: 10.1107/S1600536811033344/fy2021sup1.cif

Structure factors: contains datablock(s) I. DOI: 10.1107/S1600536811033344/fy2021Isup2.hkl

Supplementary material file. DOI: 10.1107/S1600536811033344/fy2021Isup3.cml

Additional supplementary materials:  crystallographic information; 3D view; checkCIF report

## Figures and Tables

**Fig. 1.**
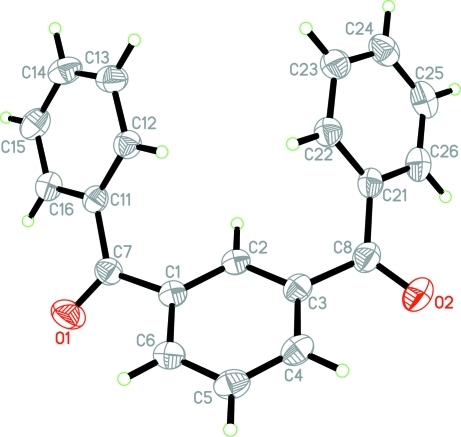
Molecular structure of title compound. Displacement ellipsoids are drawn at the 50% probability level.

## supplementary crystallographic information

### Comment

Dibenzoylbenzene represents the class of diarylketones in which a carbonyl group is present between two phenyl rings. The parent diarylketone is benzoylbenzene, which is also known as benzophenone and is a widely used as a building block in organic synthesis. Benzoylbenzene and its derivatives are important chemicals or intermediates in the dyes, pharmaceutical, pesticide and other chemical industries (Olah, 1964; Szmant, 1989; March, 1992). In the pharmaceutical industry, these are used as farnesyltransferase inhibitors (Bohm *et al.*, 2001) and non-nucleoside reverse transcriptase inhibitors of HIV-1 (Chan *et al.*, 2004) and are renowned to be effective anesthetics (Husain *et al.*, 2006) and the strongest photosensitizer among non-steroidal anti-inflammatory drugs (Bagheri *et al.*, 2000). In the fragrance industry, benzoylbenzene is a useful additive in perfumes, colognes and scented soaps. Symmetrical and unsymmetrical benzoylbenzenes functionalized with electron-donating or withdrawing groups are found in a large number of plants of the Guttiferae family (Baggett *et al.*, 2005; Chiang *et al.*, 2003). In the past few decades, numerous natural products bearing a benzoylbenzene architecture have been reported such as cariphenones A and B (Bernardi *et al.*, 2005), balanol (Kulanthaivel *et al.*, 1993), and pestalone (Iijima *et al.*, 2004). The chemistry of symmetrical and unsymmetrical benzoylbenzene includes many synthetic methods. Generally benzoylbenzene and its derivatives are prepared *via* Friedel–Crafts acylation of aromatic compounds catalyzed by Lewis acids, such as AlCl_3_, BF_3_, TiCl_4_, or ZnCl_2_ (Karrer *et al.*, 2000; Kowalski *et al.*, 2005). The title compound was synthesized successfully in an attempt to prepare dibenzoylbenzene compounds.

### Experimental

For the synthesis of 1,3-dibenzoylbenzene, a 250 ml three-necked round bottomed flask equipped with a thermometer and a magnetic stirrer was charged with 20 milliliters of benzene and 19 g (0.15 mole) of anhydrous aluminium chloride (AlCl_3_). Then 9 g (0.044 mole) of isophathaloyl chloride was gradually added into the flask over a period of 2 h. During this addition, the temperature of the reaction mixture was maintained at 285–291 K. After the addition was complete, the reaction was continued at 291 K for another 4 h. The mixture was slowly heated to 313 K and kept at that temperature for 2h. Finally, the reaction mixture was cooled and poured into 200 ml of aqueous HCl solution. Some white solid precipitated out, which was filtered, washed with ethanol and the crude product obtained was recrystallized from petroleum ether (b.p. 333–363 K). The related yield is 80% and melting point of the product is 378 K. For the growth of single crystals the compound was dissolved in petroleum ether (b.p. 333–363 K) and set aside for crystallization.

### Refinement

H atoms were geometrically positioned and refined using a riding model with C—H = 0.95Å and U(H) set to 1.2*U*_eq_(C).

### Crystal data

**Table:**

C_20_H_14_O_2_	*F*(000) = 1200
*M**_r_* = 286.31	*D*_x_ = 1.307 Mg m^−^^3^
Orthorhombic, *P**b**c**a*	Mo *K*α radiation, λ = 0.71073 Å
Hall symbol: -P 2ac 2ab	Cell parameters from 32870 reflections
*a* = 16.2029 (5) Å	θ = 2.7–28.7°
*b* = 7.8648 (4) Å	µ = 0.08 mm^−^^1^
*c* = 22.8422 (8) Å	*T* = 173 K
*V* = 2910.8 (2) Å^3^	Block, colourless
*Z* = 8	0.45 × 0.45 × 0.43 mm

### Data collection

**Table:**

Stoe IPDS II two-circle diffractometer	3095 reflections with *I* > 2σ(*I*)
Radiation source: fine-focus sealed tube	*R*_int_ = 0.050
graphite	θ_max_ = 28.4°, θ_min_ = 3.0°
ω scans	*h* = −21→21
40513 measured reflections	*k* = −10→10
3653 independent reflections	*l* = −29→30

### Refinement

**Table:**

Refinement on *F*^2^	Secondary atom site location: difference Fourier map
Least-squares matrix: full	Hydrogen site location: inferred from neighbouring sites
*R*[*F*^2^ > 2σ(*F*^2^)] = 0.037	H-atom parameters constrained
*wR*(*F*^2^) = 0.101	*w* = 1/[σ^2^(*F*_o_^2^) + (0.0547*P*)^2^ + 0.4425*P*] where *P* = (*F*_o_^2^ + 2*F*_c_^2^)/3
*S* = 1.05	(Δ/σ)_max_ = 0.001
3653 reflections	Δρ_max_ = 0.28 e Å^−^^3^
200 parameters	Δρ_min_ = −0.15 e Å^−^^3^
0 restraints	Extinction correction: *SHELXL97* (Sheldrick, 2008), Fc^*^=kFc[1+0.001xFc^2^λ^3^/sin(2θ)]^-1/4^
Primary atom site location: structure-invariant direct methods	Extinction coefficient: 0.0128 (12)

### Special details

**Table:**

Geometry. All e.s.d.'s (except the e.s.d. in the dihedral angle between two l.s. planes) are estimated using the full covariance matrix. The cell e.s.d.'s are taken into account individually in the estimation of e.s.d.'s in distances, angles and torsion angles; correlations between e.s.d.'s in cell parameters are only used when they are defined by crystal symmetry. An approximate (isotropic) treatment of cell e.s.d.'s is used for estimating e.s.d.'s involving l.s. planes.
Refinement. Refinement of *F*^2^ against ALL reflections. The weighted *R*-factor *wR* and goodness of fit *S* are based on *F*^2^, conventional *R*-factors *R* are based on *F*, with *F* set to zero for negative *F*^2^. The threshold expression of *F*^2^ > σ(*F*^2^) is used only for calculating *R*-factors(gt) *etc*. and is not relevant to the choice of reflections for refinement. *R*-factors based on *F*^2^ are statistically about twice as large as those based on *F*, and *R*- factors based on ALL data will be even larger.

### Fractional atomic coordinates and isotropic or equivalent isotropic displacement parameters (Å^2^)

**Table:**

	*x*	*y*	*z*	*U*_iso_*/*U*_eq_	
O1	0.57553 (5)	0.26790 (13)	0.50072 (4)	0.0480 (2)	
O2	0.72489 (5)	0.52781 (15)	0.23112 (4)	0.0539 (3)	
C1	0.60337 (6)	0.42835 (12)	0.41666 (4)	0.0262 (2)	
C2	0.60051 (6)	0.42295 (12)	0.35545 (4)	0.0259 (2)	
H2	0.5597	0.3564	0.3363	0.031*	
C3	0.65756 (6)	0.51526 (13)	0.32241 (4)	0.0286 (2)	
C4	0.71769 (6)	0.61245 (13)	0.35094 (5)	0.0334 (2)	
H4	0.7570	0.6743	0.3286	0.040*	
C5	0.72029 (7)	0.61901 (13)	0.41153 (5)	0.0347 (2)	
H5	0.7607	0.6866	0.4306	0.042*	
C6	0.66382 (6)	0.52687 (13)	0.44426 (5)	0.0304 (2)	
H6	0.6662	0.5306	0.4858	0.036*	
C7	0.54757 (6)	0.32464 (13)	0.45492 (4)	0.0293 (2)	
C8	0.65929 (7)	0.50879 (14)	0.25675 (5)	0.0338 (2)	
C11	0.45990 (6)	0.29171 (12)	0.43842 (4)	0.0268 (2)	
C12	0.41725 (6)	0.38926 (13)	0.39722 (4)	0.0303 (2)	
H12	0.4445	0.4802	0.3778	0.036*	
C13	0.33493 (7)	0.35381 (15)	0.38441 (5)	0.0365 (2)	
H13	0.3063	0.4198	0.3561	0.044*	
C14	0.29496 (7)	0.22188 (15)	0.41305 (6)	0.0400 (3)	
H14	0.2388	0.1978	0.4044	0.048*	
C15	0.33652 (7)	0.12507 (14)	0.45422 (5)	0.0378 (3)	
H15	0.3089	0.0347	0.4736	0.045*	
C16	0.41827 (6)	0.15987 (13)	0.46714 (5)	0.0312 (2)	
H16	0.4463	0.0938	0.4957	0.037*	
C21	0.58125 (6)	0.47839 (13)	0.22351 (4)	0.0300 (2)	
C22	0.50446 (7)	0.53230 (13)	0.24428 (5)	0.0313 (2)	
H22	0.5006	0.5873	0.2812	0.038*	
C23	0.43365 (7)	0.50573 (15)	0.21107 (5)	0.0371 (2)	
H23	0.3815	0.5417	0.2254	0.044*	
C24	0.43937 (8)	0.42670 (15)	0.15702 (5)	0.0400 (3)	
H24	0.3910	0.4086	0.1344	0.048*	
C25	0.51537 (9)	0.37388 (15)	0.13580 (5)	0.0414 (3)	
H25	0.5190	0.3199	0.0987	0.050*	
C26	0.58596 (8)	0.39992 (15)	0.16880 (5)	0.0375 (3)	
H26	0.6380	0.3641	0.1541	0.045*	

### Atomic displacement parameters (Å^2^)

**Table:**

	*U*^11^	*U*^22^	*U*^33^	*U*^12^	*U*^13^	*U*^23^
O1	0.0428 (5)	0.0614 (6)	0.0398 (5)	−0.0123 (4)	−0.0126 (4)	0.0191 (4)
O2	0.0303 (4)	0.0890 (7)	0.0425 (5)	−0.0035 (4)	0.0115 (4)	0.0008 (5)
C1	0.0234 (4)	0.0247 (4)	0.0305 (5)	0.0028 (3)	−0.0006 (4)	−0.0009 (4)
C2	0.0228 (4)	0.0243 (4)	0.0307 (5)	0.0014 (3)	−0.0005 (4)	−0.0021 (4)
C3	0.0241 (5)	0.0278 (5)	0.0338 (5)	0.0032 (4)	0.0029 (4)	−0.0005 (4)
C4	0.0251 (5)	0.0303 (5)	0.0448 (6)	−0.0021 (4)	0.0041 (4)	−0.0002 (4)
C5	0.0273 (5)	0.0323 (5)	0.0444 (6)	−0.0034 (4)	−0.0023 (4)	−0.0075 (4)
C6	0.0268 (5)	0.0308 (5)	0.0336 (5)	0.0027 (4)	−0.0029 (4)	−0.0049 (4)
C7	0.0290 (5)	0.0301 (5)	0.0287 (5)	−0.0004 (4)	−0.0017 (4)	0.0011 (4)
C8	0.0283 (5)	0.0387 (5)	0.0344 (5)	0.0019 (4)	0.0063 (4)	0.0018 (4)
C11	0.0256 (5)	0.0274 (4)	0.0273 (4)	0.0014 (4)	0.0024 (3)	−0.0031 (4)
C12	0.0285 (5)	0.0316 (5)	0.0310 (5)	0.0020 (4)	0.0018 (4)	0.0003 (4)
C13	0.0298 (5)	0.0404 (6)	0.0393 (6)	0.0054 (4)	−0.0045 (4)	−0.0026 (5)
C14	0.0268 (5)	0.0412 (6)	0.0520 (7)	−0.0019 (4)	−0.0020 (5)	−0.0085 (5)
C15	0.0321 (5)	0.0326 (5)	0.0487 (6)	−0.0049 (4)	0.0081 (5)	−0.0024 (5)
C16	0.0311 (5)	0.0285 (5)	0.0340 (5)	0.0014 (4)	0.0039 (4)	0.0002 (4)
C21	0.0314 (5)	0.0305 (5)	0.0282 (5)	−0.0004 (4)	0.0046 (4)	0.0032 (4)
C22	0.0315 (5)	0.0325 (5)	0.0299 (5)	0.0023 (4)	0.0019 (4)	0.0011 (4)
C23	0.0328 (5)	0.0389 (6)	0.0394 (6)	−0.0002 (4)	−0.0020 (4)	0.0079 (5)
C24	0.0467 (7)	0.0364 (6)	0.0369 (5)	−0.0107 (5)	−0.0092 (5)	0.0086 (5)
C25	0.0601 (8)	0.0362 (6)	0.0278 (5)	−0.0078 (5)	0.0007 (5)	0.0007 (4)
C26	0.0435 (6)	0.0388 (6)	0.0302 (5)	0.0000 (5)	0.0097 (4)	0.0011 (4)

### Geometric parameters (Å, °)

**Table:**

O1—C7	1.2242 (12)	C12—H12	0.9500
O2—C8	1.2227 (13)	C13—C14	1.3870 (17)
C1—C6	1.3989 (14)	C13—H13	0.9500
C1—C2	1.3995 (13)	C14—C15	1.3847 (17)
C1—C7	1.4990 (14)	C14—H14	0.9500
C2—C3	1.3969 (14)	C15—C16	1.3844 (15)
C2—H2	0.9500	C15—H15	0.9500
C3—C4	1.3993 (15)	C16—H16	0.9500
C3—C8	1.5009 (15)	C21—C26	1.3958 (15)
C4—C5	1.3857 (16)	C21—C22	1.3975 (14)
C4—H4	0.9500	C22—C23	1.3913 (16)
C5—C6	1.3861 (15)	C22—H22	0.9500
C5—H5	0.9500	C23—C24	1.3852 (17)
C6—H6	0.9500	C23—H23	0.9500
C7—C11	1.4923 (14)	C24—C25	1.3870 (19)
C8—C21	1.4942 (15)	C24—H24	0.9500
C11—C12	1.3970 (14)	C25—C26	1.3851 (18)
C11—C16	1.4002 (14)	C25—H25	0.9500
C12—C13	1.3937 (15)	C26—H26	0.9500
			
C6—C1—C2	119.36 (9)	C14—C13—C12	119.83 (10)
C6—C1—C7	117.45 (9)	C14—C13—H13	120.1
C2—C1—C7	123.09 (9)	C12—C13—H13	120.1
C3—C2—C1	120.13 (9)	C15—C14—C13	120.31 (10)
C3—C2—H2	119.9	C15—C14—H14	119.8
C1—C2—H2	119.9	C13—C14—H14	119.8
C2—C3—C4	119.54 (9)	C16—C15—C14	120.09 (10)
C2—C3—C8	122.32 (9)	C16—C15—H15	120.0
C4—C3—C8	118.09 (9)	C14—C15—H15	120.0
C5—C4—C3	120.44 (10)	C15—C16—C11	120.49 (10)
C5—C4—H4	119.8	C15—C16—H16	119.8
C3—C4—H4	119.8	C11—C16—H16	119.8
C4—C5—C6	119.95 (10)	C26—C21—C22	119.12 (10)
C4—C5—H5	120.0	C26—C21—C8	118.65 (10)
C6—C5—H5	120.0	C22—C21—C8	122.17 (9)
C5—C6—C1	120.57 (10)	C23—C22—C21	120.23 (10)
C5—C6—H6	119.7	C23—C22—H22	119.9
C1—C6—H6	119.7	C21—C22—H22	119.9
O1—C7—C11	120.32 (9)	C24—C23—C22	119.88 (11)
O1—C7—C1	118.25 (9)	C24—C23—H23	120.1
C11—C7—C1	121.42 (8)	C22—C23—H23	120.1
O2—C8—C21	120.79 (10)	C23—C24—C25	120.34 (11)
O2—C8—C3	119.38 (10)	C23—C24—H24	119.8
C21—C8—C3	119.83 (9)	C25—C24—H24	119.8
C12—C11—C16	118.95 (9)	C26—C25—C24	119.91 (10)
C12—C11—C7	123.07 (9)	C26—C25—H25	120.0
C16—C11—C7	117.95 (9)	C24—C25—H25	120.0
C13—C12—C11	120.33 (10)	C25—C26—C21	120.50 (11)
C13—C12—H12	119.8	C25—C26—H26	119.8
C11—C12—H12	119.8	C21—C26—H26	119.8
			
C6—C1—C2—C3	−0.02 (14)	C1—C7—C11—C16	163.41 (9)
C7—C1—C2—C3	−176.19 (9)	C16—C11—C12—C13	−0.95 (15)
C1—C2—C3—C4	0.26 (14)	C7—C11—C12—C13	−178.93 (10)
C1—C2—C3—C8	177.63 (9)	C11—C12—C13—C14	0.51 (16)
C2—C3—C4—C5	−0.73 (15)	C12—C13—C14—C15	−0.12 (17)
C8—C3—C4—C5	−178.22 (9)	C13—C14—C15—C16	0.18 (17)
C3—C4—C5—C6	0.96 (16)	C14—C15—C16—C11	−0.63 (16)
C4—C5—C6—C1	−0.72 (16)	C12—C11—C16—C15	1.01 (15)
C2—C1—C6—C5	0.25 (15)	C7—C11—C16—C15	179.09 (10)
C7—C1—C6—C5	176.64 (9)	O2—C8—C21—C26	27.69 (16)
C6—C1—C7—O1	−32.33 (14)	C3—C8—C21—C26	−151.99 (10)
C2—C1—C7—O1	143.91 (11)	O2—C8—C21—C22	−149.55 (12)
C6—C1—C7—C11	147.13 (9)	C3—C8—C21—C22	30.77 (15)
C2—C1—C7—C11	−36.62 (14)	C26—C21—C22—C23	0.87 (15)
C2—C3—C8—O2	−149.63 (11)	C8—C21—C22—C23	178.10 (10)
C4—C3—C8—O2	27.79 (16)	C21—C22—C23—C24	−0.48 (16)
C2—C3—C8—C21	30.05 (15)	C22—C23—C24—C25	−0.01 (17)
C4—C3—C8—C21	−152.53 (10)	C23—C24—C25—C26	0.11 (17)
O1—C7—C11—C12	160.85 (11)	C24—C25—C26—C21	0.30 (17)
C1—C7—C11—C12	−18.60 (15)	C22—C21—C26—C25	−0.78 (16)
O1—C7—C11—C16	−17.14 (15)	C8—C21—C26—C25	−178.11 (10)
